# A Multi-AUV Maritime Target Search Method for Moving and Invisible Objects Based on Multi-Agent Deep Reinforcement Learning

**DOI:** 10.3390/s22218562

**Published:** 2022-11-07

**Authors:** Guangcheng Wang, Fenglin Wei, Yu Jiang, Minghao Zhao, Kai Wang, Hong Qi

**Affiliations:** 1 College of Computer Science and Technology, Jilin University, Changchun 130012, China; 2 State Key Lab of Symbolic Computation and Knowledge Engineering of Ministry of Education, Jilin University, Changchun 130012, China

**Keywords:** multi-agent reinforcement learning, maritime floating objects, target search, autonomous underwater vehicles (AUV)

## Abstract

Target search for moving and invisible objects has always been considered a challenge, as the floating objects drift with the flows. This study focuses on target search by multiple autonomous underwater vehicles (AUV) and investigates a multi-agent target search method (MATSMI) for moving and invisible objects. In the MATSMI algorithm, based on the multi-agent deep deterministic policy gradient (MADDPG) method, we add spatial and temporal information to the reinforcement learning state and set up specialized rewards in conjunction with a maritime target search scenario. Additionally, we construct a simulation environment to simulate a multi-AUV search for the floating object. The simulation results show that the MATSMI method has about 20% higher search success rate and about 70 steps shorter search time than the traditional search method. In addition, the MATSMI method converges faster than the MADDPG method. This paper provides a novel and effective method for solving the maritime target search problem.

## 1. Introduction

The development of Autonomous Underwater Vehicles (AUVs) has permitted the automatization of many tasks initially achieved with human-crewed vehicles in underwater environments. Target search is the search for floating objects in the water or seafloor, considered one of AUVs’ most important tasks. Xiang et al. [1,2] propose an effective strategy for multi-AUV target search in 3-D underwater environments with obstacles and offer an integrated algorithm for a cooperative team of multiple autonomous underwater vehicles. Li et al. [3] propose an improved, rapidly exploring random trees algorithm for the AUV target search problem. However, since floating objects can move with ocean flows, AUVs need more time and better strategies for target search. Take flight accidents as an example. The first thing is to find the aircraft’s wreckage, which helps to look for the black boxes. In the case of the disappearance of Malaysia Airlines Flight 370, the crash damaged the GPS of the plane. Several countries spent a lot of workforce and resources but found no plane wreckage. After the golden period of search and rescue, the chances of finding the wreckage became less and less with the irregular movement of the ocean currents.

AUV’s sensors play a crucial role in target search, and Koopman meticulously investigates the effect of sensors on target search [4]. Modern AUVs are complex robotic systems containing several proprioceptive sensors, such as compasses, fiber optic gyroscopes, and Doppler velocity recorders. The resulting sensor outputs can be combined with navigation filters, such as an extended Kalman filter, to produce a high-quality estimate of the AUV’s position and uncertainty [5]. Side-scan sonar, initially developed by the US Navy, has become a central underwater sensor with many applications, such as detecting the seafloor, searching, and rescuing. The sector scanning sonar was considered over other acoustic alternatives such as echosounders and multibeam as a means for object detection for small AUVs [6].

There are few studies on multi-agent target search. Many researchers have carried out some attempts at multi-agent search and rescue for moving objects at sea. Target trajectory prediction is considered a significant step for some target search methods. The fragments drift on the ocean without power when the plane is shipwrecked. In this case, various environmental factors can affect its drift trajectory, such as wind, shallow currents, and waves [7]. Therefore, combining meteorological data and ocean current data to predict the drift trajectory of the target can improve the success rate of the target search and shorten the search time. Xiong et al. [8] developed a three-stage decision support approach to optimize the allocation of search and rescue resources to shorten the response time. Ai et al. [9] considered comprehensive search and rescue coverage and considered obstacles at sea, planning a search path that took the shortest time to be the safest and prioritized coverage of critical areas. Many search and rescue studies also design search systems consisting of multiple search and rescue vehicles to enhance search efficiency [10,11,12]. However, these search methods require certain location information, and trajectory prediction becomes highly challenging when the target’s initial position is unidentified.

Searching for moving objects, like plane fragments, is usually complicated due to an unknown initial position (usually, the GPS device is damaged). There are also many other crucial issues in searching for moving objects in the sea, such as insufficient information, large search areas, long search time, logistical difficulties, and harsh marine environments. Scharff et al. [13] investigated AUV path planning methods in underwater 3D environments. Meghjani et al. [14] tested the traditional outward and inward spiral methods for searching. However, the performance of these methods dramatically declines when the moving speed of the drifting object is fast, or the exploration area is large. Therefore, there is an urgent need for an efficient multi-agent target-oriented search and path planning method. 

Single-agent deep reinforcement learning methods have been widely applied to industrial fields, such as autonomous driving and unmanned aerial vehicle [15,16,17,18]. Researchers have also made some breakthroughs in multi-agent deep reinforcement learning methods [19,20]. Multi-agent reinforcement learning algorithms to specialized fields with promising results. Jiang et al. [21] propose a value-iteration-based RL algorithm, which can efficiently converge to stable strategies and significantly improve network performance. Jo et al. [22] propose a multi-agent Deep Q-learning (DQL)-based transmission power control algorithm which minimizes the outage probability of the High-Altitude Platform Station downlink. However, existing multi-agent algorithms are capable of static target search tasks and have poor performance for moving and invisible objects. Traditional spiral algorithms have fixed path patterns, which are not applicable to moving targets. Existing deep reinforcement methods lack the learning of historical information, resulting in many duplicate routes. We add map information to the multi-agents’ target search algorithm to reduce the duplicate routes and decrease the search time.

This study proposes a multi-AUV maritime target search method for the target search problem of moving and invisible objects (MATSMI). MATSMI incorporates the spatial-temporal information of the exploration map into the reinforcement learning state based on MADDPG [23]. MATSMI record the agents’ detection trajectory on an exploration map, which is one of the bases for decision-making and network critique. Furthermore, we construct a multi-AUV maritime target search simulation environment for multi-agent reinforcement learning. The average search success rate (ASSR) and the average search time (AST) are selected as evaluation metrics to evaluate the effectiveness of the proposed method. The simulation results show that the ASSR and AST of MATSMI are better than traditional search methods. At the same time, the MATSMI algorithm has dramatically improved the convergence speed compared to the original MADDPG algorithm. 

The rest of this paper is organized as follows. Section 2 presents the related work about the maritime search problem. Section 3 introduces the mechanism of the MATSMI algorithm. Section 4 shows simulation experiments for various situations. Finally, we summarize the conclusions and future work in Section 5.

## 2. Related Work

### 2.1. Target Search

In the 1970s, several scholars conducted in-depth studies on target search. Stone et al. [24] developed algorithms for arbitrary discrete time target motions and exponential detection functions. Stone et al. [25] found necessary and sufficient conditions for optimal detection problems involving regular detection functions and an essentially arbitrary stochastic process for the target motion. Washburn [26] generalized Brown’s algorithm to the class of forward and backward algorithm that apply to a more general class of payoff functions. Algorithms for non-Markovian motions are given by Stromquist and Stone [27].

Research on visible target search is well-developed, and many path-planning solutions exist. Li, J et al. [28] propose an adaptive real-time path planning method based on Deep Reinforcement Learning which accelerates the algorithm’s convergence speed and enhances the planned path’s smoothness. Yu et al. [29] proposed a hybrid multi-target path planning algorithm that improves search speed and smooths the planning path. In addition, the method has strong applicability and high effectiveness. Nussbaum et al. [30] introduce the Moving Target Search method with Subgoal Graphs. The algorithm optimizes the agent’s knowledge during the search and meets the requirement of real-time performance. Botea et al. [31] use compressed path databases in moving target searches whose results are orders of magnitude better than the state-of-the-art methods. 

Many researchers focus on target search where the target is not visible. Song et al. [32] propose a two-stage optimization model for path planning for the target search of mobile robots. Moreover, they proposed A method for determining complete visual coverage of critical locations on a 2D grid map. Farzad Niroui et al. [33] combine traditional exploration methods with deep reinforcement learning to enable robots to explore unknown cluttered environments autonomously. Liu Z et al. [34] investigated a collaborative search and coverage algorithm for a given bounded rectangular region by a set of Unmanned Aerial Vehicles. They construct a cognitive map including information such as target probabilities as a representation of the environment; and establish a revisit mechanism. McCalmon J [35] proposed a method for efficiently exploring unknown regions with accurate coverage of regions of interest. Walker O et al. [36] proposed a multi-agent target-finding framework based on online POMDP planning and deep reinforcement learning control.

Multi-agent systems have made great strides in self-organizing mesh networks and have achieved high levels of reliability and security in communications. Although many researchers are gradually progressing in several vital areas, it has yet to produce perfect results in search and rescue [37].

### 2.2. Spiral Search Method

In the spiral search method, the agent uses a spiral trajectory to cover the target area to search for the target [14]. The interval of each circle of this trajectory is the detection diameter of the agent, which can ensure the full coverage of the area search. In the limiting case, where the floating object moves in the direction of the radius of the spiral trajectory, the algorithm needs to ensure that the target cannot leave this detection ring after one revolution of the agent’s search. As such, the spiral search algorithm will ensure that the agent can find the target. 

The algorithm performs relatively well when searching for stationary targets or targets at low speeds or miniature ranges. However, it is not easy to have excellent results when the movement range is more extensive, or the target moves faster.

### 2.3. MADDPG Framework

The MADDPG algorithm is a generalized multi-agent deep reinforcement learning algorithm [23]. Extend the single-agent Deep Deterministic Policy Gradient (DDPG) algorithm obtaining this algorithm, where each agent has its network with no central control. The tasks are performed together only by collaborating. Its network is also composed of two parts: the actor and the critic. The actor selects actions based on the state obtained from the environment. The critic evaluates the actions obtained by the actor to improve the actor’s performance. The inputs of these two networks are different. The actor’s input is its state. The critic’s input is the states and actions of all agents. The MADDPG algorithm uses a replay buffer. It saves the data generated by each executed action in strips to the replay buffer. The replay buffers randomly draw experience for training which disrupts the order of the data, resulting in a more uniform input to the network and improving the training effect.

The main innovation of this algorithm is that it feeds the states and actions of other agents into the critic during the training process, which makes the evaluation more comprehensive and allows the network to converge faster.

## 3. MATSMI Method

This section outlines a formal description of the maritime target search for moving and invisible objects problem. Moreover, we design a corresponding simulation environment for multi-agent reinforcement learning. Then we describe in detail the MATSMI in this paper.

### 3.1. Formalization of the Maritime Target Search Problem

The area scenario is a square area D. There are $N_{A}$ search AUVs. We set initial positions of AUVs according to the specific situation. The detection area of a single AUV is circular. Moreover, we defined $D_{i}(t)$ as the detection area of AUV i at the time step t.

The velocity direction of AUV is $vel_{i}$. The velocity magnitude can be chosen arbitrarily in the interval $\left[0,v_{m}\right]$. A target T appears randomly in the region D in a uniform distribution. Then move the target under the action of a random environmental force $(f_{x},f_{y})\in F_{env}$. The environmental force $\left(f_{x},f_{y}\right)$ is randomly drawn from historical ocean wind, shallow currents, and waves environmental force data $F_{env}$.

During simulation i, the target search is successful, and the variable $I_{i}$ is equal to one when the target object *T* appears in the detection area $D_{i}(t)$ of any AUV. Conversely, the search failed and $I_{i}$ was set to zero when the action time t reaches the upper limit $t_{m}$, or the target object leaves the area. There are two evaluation metrics for this problem, the *ASSR*, and the *AST*. The optimization objective of the MSTSPP algorithm is at max *ASSR* and min *AST*.

(1) $$ASSR=\frac{1}{N}\overset{N}{\underset{i}{\sum }}I_{i}$$

(2) $$AST=\frac{1}{N}\overset{N}{\underset{i}{\sum }}t_{i}$$

where N is the number of simulations.

In addition, adhere to the following constraints:

- AUVs always have to be in the region, $\forall i,j,(x_{i}^{A}(t),y_{i}^{A}(t))\in D$ at any time;
- AUVs cannot collide with each other, $\forall i,j,(x_{i}^{A}(t),y_{i}^{A}(t))\neq (x_{j}^{A}(t),y_{j}^{A}(t))$ at any time.

### 3.2. Create a Multi-Agent System

#### 3.2.1. MADDPG Algorithm Improvement

Based on the DDPG algorithm, The MADDPG exploits other agents’ state and action information. Nevertheless, some useful spatiotemporal information is not utilized, for example, past actions that a single agent cannot observe. Following the idea above, we record the exploration trajectories traveled by the agents in the form of exploration maps in this paper. We append the exploration map to the training and decision process after processing.

In order to improve the search success rate of target search problems, it is necessary to guide the agent to expand the search area as much as possible to find the target. Moreover, the exploration trajectory of the agent can help the agent know which regions are still unexplored. We divide the area into grids, and the value of each grid represents the number of times the agent explores. When the agent explores the grid, the value is added by one. This map gathers historical exploration information of all agents. Since the target constantly moves, the likelihood of a target reappearing in an explored area becomes higher as time passes. Therefore, we set the values in the map to change back to zero after exceeding the map memory time $t_{m}$, which encourages agents to revisit the explored area.

After that, carry out a preallocation of the unexplored areas on the map. Assign the grid to the corresponding agent according to its distance from each agent. Once an agent acquires a map, perform this process, and regard the unexplored areas allocated to other agents as explored. It reduces the difficulty of cooperation between agents. 

It will consume many computational resources to allow agents to use the whole map to make decisions. The most effective basis for decision-making is the map information around the agent, meaning that many parts of the map are redundant. Hence, each agent only needs to access the map within a certain distance of his current coordinates. In this paper, this distance is called map visual distance $l_{m}$.

Further, we simplify the map to help agents learn the information more quickly. Firstly, convert the map matrix into a square matrix centred on the current coordinates of the agent. Secondly, accumulate the values of matrices with distances less than $l_{m}$ in eight directions, which are at 0 or 45 degrees from the coordinate axis. Therefore, we convert the map matrix to values for the degree of exploration in the eight directions around the current agent, making learning less challenging and faster converging.

#### 3.2.2. Multi-Agent System of MATSMI

With the formalization of the target search problem in Section 3.1, we can create a multi-agent reinforcement learning system. In this system, each AUV is abstracted to an agent. 

There are $N_{A}$ agents in the system. Each agent has a set of deterministic policies $\mu =\{\mu_{\theta_{1}},\mu_{\theta_{2}},\ldots ,\mu_{\theta_{N_{U}}}\}$ for selecting actions. In addition, it has a set of critic networks.

The loss function of the critic network is as follows:

(3) $$L(\theta_{i})=E_{x,m,a,r,x^{\prime },m^{\prime }}[{\left(Q_{i}^{\mu }(x,m,a_{1}\ldots ,a_{N})-y\right)}^{2}],$$

(4) $$y=r_{i}+\gamma Q_{i}^{\mu^{\prime }}(x^{\prime },m^{\prime },{a^{\prime }}_{1},\ldots ,{a^{\prime }}_{N}){|}_{{a^{\prime }}_{j}={\mu^{\prime }}_{j}(o_{j},m_{j})}$$

The strategy gradient of the actor network is as follows:

(5) $$\nabla_{\theta_{i}}J(\mu_{i})=E_{x,a,m\in D}[\nabla_{\theta_{i}}\mu_{i}(a_{i}|o_{i},m_{i})\nabla_{a_{i}}Q_{i}^{\mu }(x,a,m){|}_{a_{i}=\mu_{i}(o_{i},m_{i})}]$$

where:

$\theta_{i}$ is the neural network parameter of the agent. 

x is a set of current states. $x=\{o_{1},o_{2},\ldots ,o_{N_{A}}\}$

m is the current map information.

$x^{\prime }$ is the next step states.

$m^{\prime }$ is the next step map information.

$Q_{i}^{\mu }(x,a,m)$ is the Q function.

$Q_{i}^{\mu^{\prime }}(x^{\prime },m^{\prime },a^{\prime })$ is the target Q function which updates towards the Q function within a specific range.

y is the predicted Q value obtained by the target Q function.

$a_{i}$ is the action of the agent i.

$o_{i}$ is the current agent’s observation i, containing the coordinates of all agents.

$m_{i}$ is the current map information of the agent i.

$r_{i}$ is the reward that the agent i receives after performing the current action.

γ is the discount value of deep reinforcement learning.

D is the replay buffer.

$\left(x,m,x^{\prime },m^{\prime },a,r\right)$ is a record in the replay buffer containing all the experience gained by the agent in a single execution.

This multi-agent system can train the network in a simulated environment and optimize the final target to obtain the desired results.

### 3.3. Reward Setting of MATSMI

The goal of the MATSMI algorithm is to maximize ASSR and minimize AST. Therefore, it needs to design suitable rewards for achieving this optimization goal. The core of the target search problem is to find the target. When an agent discovers the target, give a grand reward $R_{target}$ to encourage searching for the target. In addition, the quicker the discovery, the higher the reward, encouraging agents to find the target as quickly as possible. 

The agent’s continuous action space makes the algorithm’s convergence relatively tricky. Therefore, we need to restrict the actions’ degrees of freedom. Here, we design the wall-bumping penalty $R_{punish}$ to restrict the exploration area of the agent. 

The agent has difficulty learning the correspondence between the state and the discovery target. To help agents learn this correspondence, we design inspirational rewards that promote exploration. When the agent explores a new area, we give a positive reward. When it repeatedly explores a region, we penalize it. In this way, agents are encouraged to explore new areas. As a result, agents can better learn the connection between the state and the discovery target. At the same time, this helps agents to cooperate with other agents by giving rewards based on the shared exploration maps of all agents.

### 3.4. MATSMI Architecture and Algorithm

**Figure 1:**
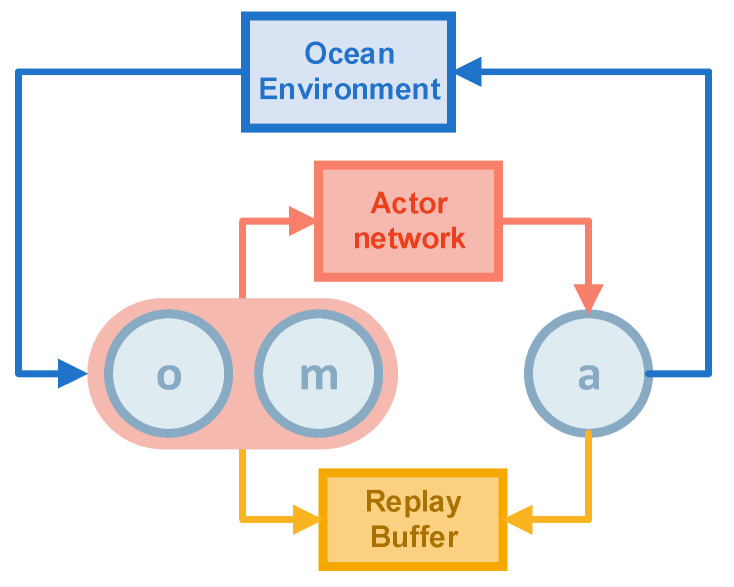
The execution process of MATSMI.

**Figure 2:**
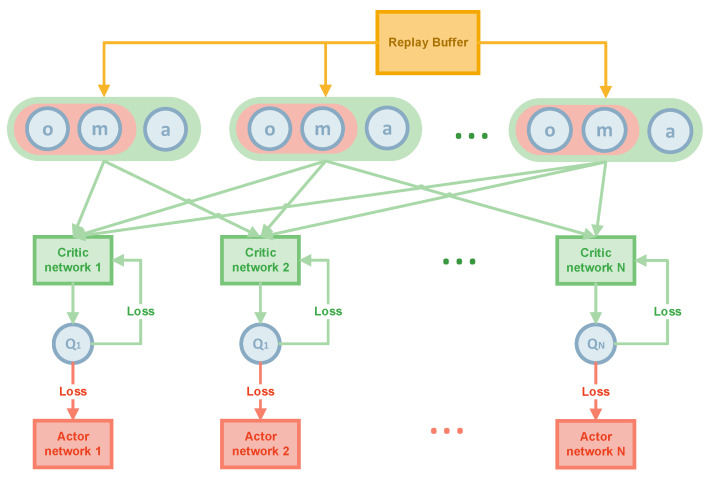
The training process of MATSMI.

The Algorithm 1 aims to solve the target search problem for moving and invisible objects. The execution and training processes are shown in Figure 1 and Figure 2.

**Algorithm 1** MATSMI1.Set epochs, $N_{A}$2.Initialize the actor network parameter and the critic network parameter3.**for**j←1 to epochs **do**4.  Reset env5.  **for** i←1 **to** $N_{A}$ **do**6.    Choose an action based on the policy $u_{i}=\mu_{\theta_{i}}(o_{i},m_{i})+N$7.    Obtain rewards r(t+1)**,** s(t+1) and m(t+1) as a result of the action $u_{i}$8.    Store the experience $<o,u,r,m,m^{\prime },o^{\prime }>$ to replay buffer D9.  **end for**10.  **if** j>batch size **then**11.    Sample a minibatch $<o,u,r,m,m^{\prime },o^{\prime }>$ from replay buffer D12.    **for** i←1 **to** $N_{A}$ **do**13.      Compute the policy gradient $\nabla_{\theta_{i}}j(\mu_{i})$ to update Actor network14.      Minimizing the loss function $L(\theta_{i})$ to update Critic network15.      Soft update the target network according to the existing critical network16.    **end for**17.  **end if**18.**end for**
where:

$u_{i}$ is the determined action of the agent i.

<o,u,r,m,m’,o’> is a record in the replay buffer containing all the experience gained by the agent in a single execution. 

Each agent in the algorithm has an actor network (the red rectangular in Figure 1 and Figure 2), a critic network (the green rectangular in Figure 1 and Figure 2), and a target critic network. The target critic network is a copy of the critic network to optimize the loss. Subsequently, soft update the target critic network toward the critic network whenever the critic network is updated, which reduces the magnitude of the update of the target critic network and makes the target critic network close to the critic network.

The algorithm has two main parts: execution and training. The first is the execution part. The state and map information is first obtained from the environment and then fed into the actor network to obtain the action. Then the action is executed in the simulated environment to obtain the new state and map information. We stored the experience generated in a replay buffer (the orange rectangular in Figure 1 and Figure 2). a is the action of the agent, o is the observation of the agent, containing the coordinates of all agents, and m is the map information seen by the current agent (the exact definition as the Section 3.2.2). The map information is processed the same way as in Section 3.2.1 before being saved, reducing the exploration map matrix to a 1 × 8 matrix. 

The second part is the training part. Each AUV has its actor network, a critic network, a target critic network, and values (a, o, m). a is the action of the agent, o is the observation of agent, containing the coordinates of all agents, and m is the map information seen by the current agent (the exact definition as execution process and the Section 3.2.2). The data from the replay buffer is first drawn at random. Then, each agent will input the states, maps, and actions of all the agents in a time step into the critic network and get the Q value of the evaluation of the action. The actor network can then be updated by equation (3). The predicted Q value can also be obtained from the target critic network, and the critic network can be updated based on these two Q values. Finally, in the inference stage, by inputting the map and state of each step into the actor network, the actor network will output each step’s action and tell the AUV where to go. 

## 4. Experiment

### 4.1. Experiment Setup

MATSMI, MADDPG, and the spiral search algorithm use the same simulation environment we set up following the formalization in Section 3.1. The environment is spatially continuous, meaning it can locate agents and targets anywhere in the region. The size of the area is 20 km × 20 km. The environment is time-discrete, meaning the agent and target move once after each interval. In other words, the agent is stationary between moves. The discrete interval is 2 min.

This environment has four search AUVs, whose initial positions are the four corners of the region as Equation (6).

(6) $$\{\begin{array}{c} \left(x_{1}^{A}(0),y_{1}^{A}(0))=(0,0\right)\\ \left(x_{2}^{A}(0),y_{2}^{A}(0))=(x_{m},0\right)\\ \left(x_{3}^{A}(0),y_{3}^{A}(0))=(0,y_{m}\right)\\ \left(x_{4}^{A}(0),y_{4}^{A}(0))=(x_{m},y_{m}\right) \end{array}$$

In this environment, we also constructed a sonar model that scans the surroundings by continuously rotating the transducer to send a narrow fan-shaped sound beam. The working frequency of this sonar is 700 kHz. The sonar’s vertical and horizontal beam widths are 30 degrees and 3 degrees, respectively, allowing for a complete 360-degree sector scan and a maximum detection distance of 1000 m. When the AUV acquires the coordinates, we add some noise to the position coordinates to simulate the error caused by the position sensor.

In addition, the environment has one target object whose initial position is random in the region. We set the maximum movement speed of AUV to 4 m/s. The target trajectories are generated by randomly extracting the historical ocean current data to simulate the drift trajectories. We generate the historical environmental data through multiple simulations in the National Maritime Search and Rescue Support System. These trajectories are rotated and panned for data enhancement.

### 4.2. Comparison Experiments of MATSMI and Spiral Search Algorithm

We first conduct the MATSMI search experiment. We set the map memory time $t_{m}$ to 50 and set the map’s visual distance $l_{m}$ to 10. The training episodes of MATSMI is 10,000. Besides, we set the search completion time for the spiral search method as the maximum search time. 

**Figure 3:**
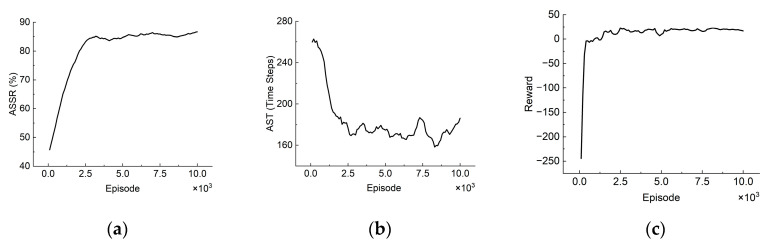
(**a**) ASSR curve, (**b**) AST curve, and (**c**) Reward curve for the MATSMI algorithm.

As shown in Figure 3, the MATSMI algorithm is close to convergence after 2500 episodes, with ASSR reaching 85% and AST reduced to 170 steps, indicating that it is feasible to apply the multi-agent reinforcement learning algorithm to the target search problem.

In the spiral search algorithm, we divide the original square area into four small squares. Each agent performs a spiral search on its small square. The four agents complete the spiral search at the same time. 

We set three target movement speeds, 1 m/s, 2 m/s, and 3 m/s, to simulate target drift in different marine environments. We can obtain more reasonable and accurate results by comparing the algorithms at different target movement speeds.

As shown in Table 1, MATSMI’s ASSR improved by nearly 20% over the spiral search for different target speeds, and MATSMI’s AST was nearly 70 steps less than the spiral search. The results indicate that the MATSMI method is more efficient than the spiral search algorithm for searching at a faster target speed.

### 4.3. Comparison Experiments of MATSMI and MADDPG

We also carried out comparative experiments between MATSMI and the original MADDPG. The MATSMI algorithm uses the map designed in Section 3.2.1, while the MADDPG algorithm uses only the coordinate information of each agent. The setup of the MATSMI algorithm is the same as in Section 4.2. These two algorithms use the same simulation environment and settings described above.

**Figure 4:**
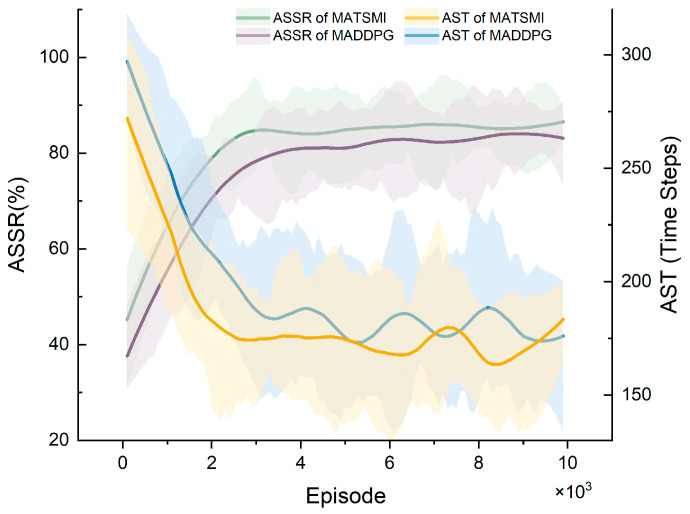
Comparison of ASSR and AST curves of MATSMI and MADDPG algorithms.

Figure 4 shows that MATSMI’s ASSR (green line) converges to 85% after 2000 episodes. In contrast, the purple line of the MADDPG algorithm only reached a similar level to the MATSMI algorithm after 9000 episodes. The AST of the MATSMI algorithm, which is the yellow line, reached 170 steps after 2000 episodes. At the same time, the MADDPG (blue line) algorithm reached the same level only after 8000 episodes. The MATSMI algorithm converges faster than the original MADDPG algorithm.

### 4.4. Search Trajectory

**Figure 5:**
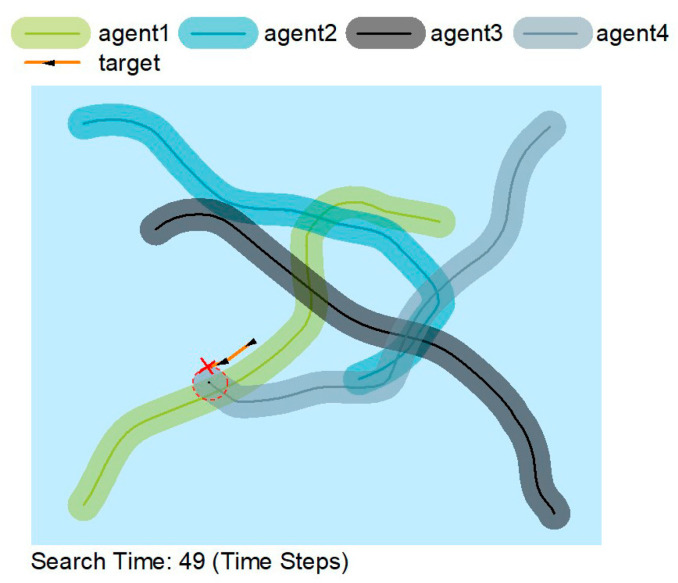
Search paths for the MATSMI algorithm.

Figure 5 shows that the agents are constantly on the move rather than searching in a constant area. The agent moves away from previous trajectories rather than near them, thus making the search more efficient by sacrificing some accuracy. AUVs can accomplish their search mission extremely well by cooperating.

**Figure 6:**
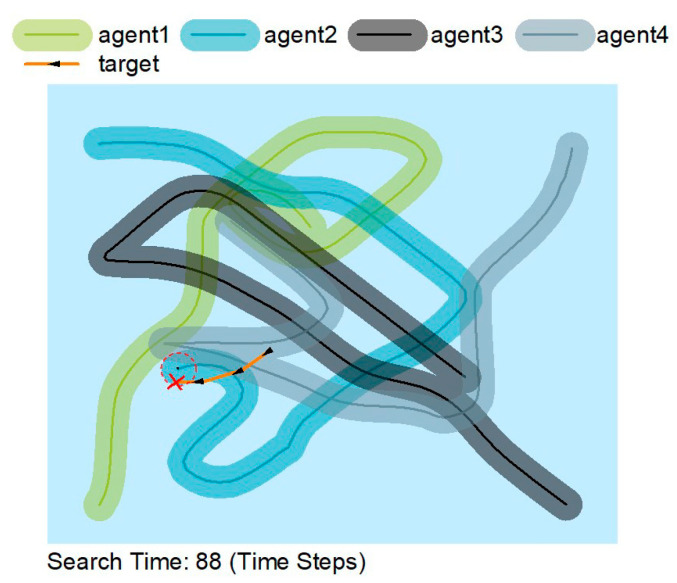
Search paths for the MADDPG algorithm.

**Figure 7:**
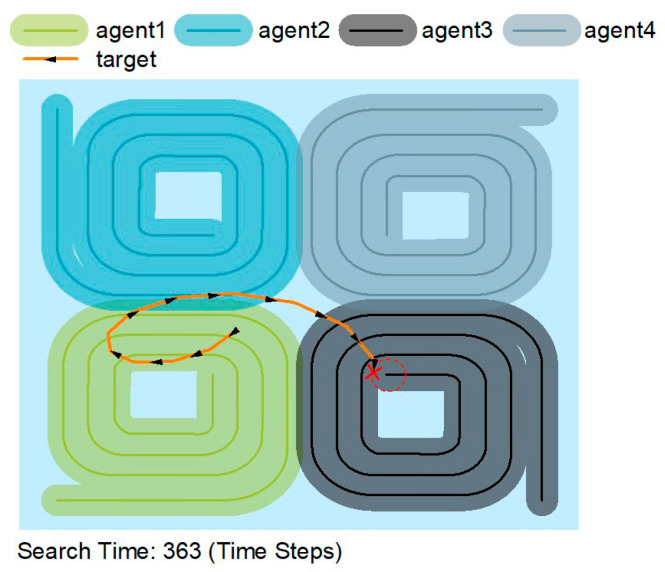
Search paths for the spiral search algorithm.

In Figure 5, Figure 6 and Figure 7, the solid lines indicate the travel trajectory of the agent, and the shading indicates the exploration area of the agent. The orange line indicates the motion trajectory of the target object. Although the orange line intersects with the green, blue, and black lines, it is only an intersection on the trajectory, and the AUV can only find the target at the red circle. Figure 5 and Figure 6 show that the MATSMI algorithm has a shorter search time of 49-time steps, which is 39-time steps less than the MADDPG algorithm for the same target motion trajectory.

Figure 7 shows that the trajectory of the spiral search algorithm is constant and searches in a spiral trajectory. The spiral search allows the search area to cover the whole map. The spiral search has the longest search time of 363-time steps when the target motion trajectory is the same.

## 5. Conclusions and Future Work

This paper applies multi-agent reinforcement learning algorithms to the maritime target search problem. Based on the MADDPG algorithm, we add map information into actor network and propose the MATSMI algorithm in conjunction with the maritime target search scenario. The experimental results show that the multi-agent reinforcement learning algorithm can solve the target search problem better than the traditional spiral search. In addition, MATSMI can converge faster compared to the original MADDPG algorithm. Moreover, this provides a new way of thinking about the maritime target search problem. In the future, we will try to extract more effective reinforcement learning states and set more reasonable rewards to allow multi-agent reinforcement learning algorithms to converge faster and with better results on target search problems. Moreover, we will consider more environmental information to simulate a more realistic environment.

## Figures and Tables

**Table 1:** ASSR and AST of MATSMI and spiral search methods at different target speeds.

Target Speed(m/s)	ASSR of MATSMI	ASSR of Spiral Search	AST of MATSMI	AST of Spiral Search
1	83.5% ± 6.5	64.5% ± 6.5	182.3 ± 20.0	255.8 ± 25.5
2	78.5% ± 4.5	61.0% ± 6.0	189.2 ± 26.0	277.1 ± 28.9
3	65.5% ± 9.5	44.5% ± 5.5	226.2 ± 29.5	292.1 ± 21.5

## Data Availability

Not applicable.
